# Effects of polystyrene nanoparticles on the microbiota and functional diversity of enzymes in soil

**DOI:** 10.1186/s12302-018-0140-6

**Published:** 2018-05-04

**Authors:** T. T. Awet, Y. Kohl, F. Meier, S. Straskraba, A.-L. Grün, T. Ruf, C. Jost, R. Drexel, E. Tunc, C. Emmerling

**Affiliations:** 1 Depart. Soil Science, Faculty of Regional and Environmental Science, University of Trier, Behringstraße 21, 54296 Trier, Germany; 2 Department of Bioprocessing and Bioanalytics, Fraunhofer Institute for Biomedical Engineering IBMT, Joseph-von-Fraunhofer-Weg 1, 66280 Sulzbach, Germany; 3 Postnova Analytics GmbH, Max-Planck Straße 14, 86899 Landsberg, Germany; 4 Institute of Molecular Biosciences, J.W. Goethe University, Max-von-Laue-Straße 9, 60438 Frankfurt am Main, Germany; 5 PlasmaChem GmbH, Schwarzschildstraße 10, 12489 Berlin, Germany; 6 Biology Department, Gaziantep University, Üniversite Bulvarı, 27310 Şehitkamil, Gaziantep, Turkey

**Keywords:** Polystyrene, Nanoparticles, Microbial biomass, Microbial activity, Enzyme activities, Environmental risk assessment

## Abstract

**Background:**

The increasing production of nanoplastics and the fragmentation of microplastics into smaller particles suggest a plausible yet unclear hazard in the natural environment, such as soil. We investigated the short-term effects (28 days) of polystyrene nanoparticles (PS-NPs) on the activity and biomass of soil microbiota, and the functional diversity of soil enzymes at environmental relevant low levels in an incubation experiment.

**Results:**

Our results showed a significant decrease in microbial biomass in treatments of 100 and 1000 ng PS-NP g^−1^ DM throughout the incubation period. Dehydrogenase activity and activities of enzymes involved in *N*-(leucine-aminopeptidase), *P*-(alkaline-phosphatase), and C-(β-glucosidase and cellobiohydrolase) cycles in the soil were significantly reduced at day 28 suggesting a broad and detrimental impact of PS-NPs on soil microbiota and enzymes. Leucine-aminopeptidase and alkaline-phosphatase activities tended to decrease consistently, while β-glucosidase and cellobiohydrolase activities increased at high concentrations (e.g., PS-NP-1000) in the beginning of the incubation period, e.g., at day 1. On the other hand, basal respiration and metabolic quotient increased with increasing PS-NP application rate throughout the incubation period possibly due to increased cell death that caused substrate-induced respiration (cryptic growth).

**Conclusions:**

We herewith demonstrated for the first time the potential antimicrobial activity of PS-NPs in soil, and this may serve as an important resource in environmental risk assessment of PS-NPs in the soil environment.

**Electronic supplementary material:**

The online version of this article (10.1186/s12302-018-0140-6) contains supplementary material, which is available to authorized users.

## Background

As a result of insufficient recycling of plastic products, millions of tons of plastics end up in landfills and oceans each year [1]. Besides polyethylene, polypropylene, polyvinylchloride, polyurethane, and polyethylenterephthalat, polystyrene (PS) is one of the most extensively used types of plastic. PS could enter the soil not only through direct release from products and applications during their life cycle [2] but also through fragmentation of macro- and microplastics. Once in the environment (e.g., landfills), polystyrene can undergo weathering through UV radiation, mechanical abrasion, biological degradation, and disintegration that result into smaller sized microplastics which could eventually fragment to nanoplastics [3]. As a result, nanoplastics are expected to increase consistently with time in the environment [4]. Recently, Lambert et al. [5] showed the formation of polystyrene nanoparticles (PS-NPs) during the degradation of PS coffee cup lids using nanoparticle tracking analysis. Moreover, there are reports of PS-degradation by microorganisms (*Rhodococcus ruber*) [6], mandibulate insects and larva of mealworms [7, 8]. Furthermore, geophagous soil fauna, particularly earthworms, could contribute to fragmentation of plastics as ingested and broken down by their gizzard. Moreover, thermal cutting of polystyrene foam has been shown to emit nano-sized polymer particles, in the range of 22–220 nm [9] which can enter the soil through atmospheric deposition.

Exposure modeling strongly suggests that soil could be a major sink of NPs compared to air and water ecosystems [10, 11]. Measuring the concentration of NPs particularly in soil, however, remains difficult due to lack of analytical methods capable of quantifying trace concentrations of NPs [12]. The absence of measured NP concentration in the environment, therefore, hampers the accuracy of eco-toxicological assessment.

In the natural environment, NPs can undergo transformation in their size, shape, charge, surface coating, agglomeration rate, density, and other properties thereby affecting their biological fate, mobility, and bioavailability [3, 13]. The small size and high surface area of NPs make it possible to pass through the biological barriers and potentially enhance their bioactivity. Nanoparticles are more inclined to cellular internalization and show more toxicity than the larger ones [14, 15]. The available studies suggest several effects of PS-NP mainly on aquatic environment; however, research on the effects of nanoplastics on soil organisms are almost rare (see also [16]). First data suggest that nano-polystyrene particles at concentrations more than 10 μg L^−1^ provoked transgenerational toxicity in the soil nematode *Caenorhabditis elegans* [17]. PS is generally considered to be non-toxic; however, PS-NPs were shown to potentially have toxic effects [16]. For example, PS-NPs were reported to have negative effects on development of sea urchin embryos, algal growth, and reproductive success of *Daphnia magna* [18]. Moreover, PS-NP transfer has been reported in a food chain from algae to zooplankton and ultimately in fish causing behavioral and metabolic changes [19]. Rossi et al. [20] showed that PS-NPs may infuse and dissolve into lipid membranes altering membrane structure and thereby potentially affecting cellular function. Similarly, [21] showed that carboxylated PS-NPs with sizes ranging from 40 to 50 nm entered cells irreversibly. Cellular uptake of carboxylic-acid functionalized PS beads was shown to be much quicker for 20 nm than 200 nm [22]. Moreover, surface modification has a profound effect on toxicity of nanoparticles. Charged NPs are more reactive towards cells and proteins compared to their neutral counterparts [23].

In this study, we investigated the short-term eco-toxicological effects of PS-NPs on the soil microbial community. Unlike toxicological studies, which use single populations in synthetic media, our study was conducted at a community level and thus, will offer an improved environmental risk assessment. Moreover, the available literature on eco-toxicological effects of NPs is mainly based on higher concentrations than would normally be expected in the natural environment [24]. Thus, our soil samples were spiked with low concentrations of 10, 100, and 1000 ng PS-NPs g^−1^ dry soil, to reflect a more realistic assessment. To assess the potential eco-toxicological effects of PS-NPs in soil, we determined soil microbiota and enzyme activities within 28 days of incubation period. These properties have been frequently referred as suitable indicators of soil health [25–28].

To our present knowledge, this is the first study of the impact of PS-NP on soil microorganisms and enzyme activities so far. We assume that nano-sized PS particles might be environmentally relevant to soil microbes and the processes they are involved in.

## Methods

### Polystyrene nanoparticles

Polystyrene nanoparticles with an unfunctionalized surface (PS-NPs) were provided by PlasmaChem (Berlin Germany) and were used as supplied. Briefly, PS-NPs were synthesized via emulsion polymerization using styrene and divinylbenzene. Rhodamine 6G was added as a fluorescence marker while addition of the surfactant sodium dodecylbenzenesulfonate (1 wt% with respect to the mass of styrene) ensured a stable emulsion. After PS-NP formation, the obtained suspension was centrifuged and the supernatant was discarded. The concentrated PS-NP suspension was prewashed with ultrapure water and then placed in an ultrafiltration tube, sealed and subsequently dialyzed with excessive ultrapure water three to five times (ratio approx. 1:20 for each dialysis step) in order to remove all water-soluble ingredients, particularly the surfactant.

### Transmission electron microscopy of PS-NPs

To investigate the appearance of the PS-NPs in soil, the soil material was diluted to a concentration of 5 mg mL^−1^ by water (HiPerSolv CHROMANORM^®^ for HPLC). This mixture was treated in an ultrasonic bath at a power of 42 W L^−1^ for 15 min, as was the PS-NP stock suspension. Afterwards, the PS-NPs were added to the soil in a concentration of 20 mg g^−1^ and the obtained suspension was shaken by hand. Afterwards, 2 µL of this suspension was applied to a single slot copper grid laminated with polyvinyl butyral and air-dried for 12 h followed by analysis in a Philips CM 12 transmission electron microscope working at an acceleration voltage of 80 kV.

### Zeta potential and size of PS-NPs

The nanoparticle surface charge (Zeta potential) was measured by Laser-Doppler-microelectrophoresis (Malvern Zetasizer Nano-ZS, Malvern Instruments Ltd., Worcestershire, United Kingdom). Sample preparation was carried out by diluting the native PS-NP suspension (63.4 mg mL^−1^) 1:10 in ultrapure water. For measurements of the Zeta potential, the diluted samples were transferred into disposable Zeta potential cuvettes (Zeta cell DTS 1060C). All measurements were carried out at a temperature of 25 °C. The Zeta potential reported herein was obtained as the average of three independent measurements (100 repetitions per measurement) performed on each sample.

The particles size was determined by dynamic light scattering (DLS) measurements in batch-mode using a Zetasizer Nano S (Malvern, UK). In detail, the native PS-NP suspension was diluted in ultrapure water (Millipore Milli-Q Integral, Billerica, USA) to a final concentration of 0.5 mg mL^−1^. Mixing was performed by thorough shaking (IKA Vibrax VXR, Staufen, Germany) for 30 s without application of ultrasonication. Size and Zeta potential of PS-NP were also measured in acidic, alkaline, and neutral environment to prove the independence of the respective pH values on PS-NP characteristics (data not shown). Six independent DLS measurements were carried out and the obtained values were averaged.

### Asymmetrical flow field-flow fractionation (AF4) of PS-NPs

AF4 measurements of the PS-NP-exposed soil samples were performed in triplicate using an AF2000 MT system (Postnova Analytics, Landsberg, Germany) consisting of an autosampler (PN5300), two isocratic pumps (PN1130) a solvent degasser (PN7520), a cartridge oven (PN4020), and an analytical fractionation cartridge equipped with a 350 µm spacer and a 10 kDa regenerated cellulose membrane. This system was hyphenated with UV/Vis-(PN3211), 21-angle multi-angle light scattering (MALS) (PN3621), and dynamic light scattering (DLS) detection (Zetasizer Nano S, Malvern, UK). Fractionation was performed using 0.05% (v/v) NovaChem (Postnova Analytics, Germany) mixed with ultrapure water as eluent. The PS-NP suspension was diluted to a final concentration of 20 µg mL^−1^. Further fractionation conditions were as follows: injection volume was adjusted to 125 µL, detector flow with 0.5 mL min^−1^, cross flow 1 mL min^−1^ with a power gradient of 0.2, injection flow 0.2 mL min^−1^ with an injection time of 7 min and a fractionation time of 50 min. A rinse step of 10 min at the end of the fractionation process was used between injections. Detailed fractionation conditions are given in the Additional file 1.

### Experimental design

The soil used for this experiment was collected at Helenenberg, NW of Trier, Germany (DD 49.8526°N, 6.5417°E) in spring 2016. Soils are deeply developed Stagnic-Luvisols derived from eolian loess. Land-use at this site was winter wheat. The experimental soil is characterized by a pH value of 7.2, amounts of total organic carbon (TOC) and total nitrogen (TN) of 28.8 and 2.0 mg g^−1^ dry mass (DM), respectively, and a subsequent C-to-N ratio of 14.4. The water-holding capacity (WHC) as well as the cation exchange capacity (CEC) of this soil are very high (Table 1). Prior to the experiment, soil was thoroughly sieved < 2 mm. For soil microbial properties, the soil moisture content was adjusted to 40% of the maximum water-holding capacity (WHCmax = 59.9%) according to [29]. The WHC of the soil was measured as stored water by percolation tests.

**Table 1:** Characterization of the soil used for the experiments

Parameters	Units	Properties
Soil type (WRB)		Stagnic-Luvisol
Soil texture		SiL (silt loam)
WHCmax	%	59.9
pH	0.01 M CaCl_2_	7.2
TOC	mg g^−1^ DM	28.8
TN	mg g^−1^ DM	2.0
C/N		14.4
Pedogenic oxides (Fe)	mg g^−1^ DM	13.4
CEC	mmol_c_ kg DM	193.3

*TOC* amount of total organic carbon, *TN* amount of total nitrogen, *C/N* carbon-to-nitrogen ratio, *CEC* cation exchange capacity, *DM* dry mass

To investigate the eco-toxicological effects of PS-NPs on soil microbial community, microbiological soil properties namely microbial biomass, respiration, and enzyme activities were assessed. Before applying the test material, the soil was moistened to a water content of 15.3%, which was equivalent to 40% WHCmax and incubated at 18 °C for 7 days. The application of the test material was performed in crystallizing dishes, each filled with soil equivalent to 100 g dry weight.

PS-NP stock suspension (63.4 mg mL^−1^) was bath sonicated at 42 W L^−1^ for 15 min and diluted in ultrapure water. Then, 1 mL PS-NP suspension, at different concentrations, was added in small drops onto the soil surface to obtain final concentrations of 10, 100, and 1000 ng g^−1^ dry weight. Negative controls only received an application of ultrapure water. For each concentration, day, and replicate, separate soil dishes were used. Each treatment was conducted in quadruplicate.

Subsequently, soils were extensively and accurately mixed by stirring with a spoon, and then transferred to glass beakers which were subsequently sealed by Parafilm^®^. They were incubated at 18 °C in the dark for 1, 14, and 28 days.

### Chemical and microbial analyses

The soil pH was determined potentiometrically in a 0.01 M CaCl_2_ solution with a glass electrode. The water content was determined after drying the soil samples at 105 °C for 24 h. Microbial biomass C (MBC) was determined by the chloroform fumigation extraction method [30]. TOC-TN analyzer (Shimadzu TOC-V + TNN) was used to determine total organic C of the extracts. For MBC analyses, a 0.01 M CaCl_2_ solution was used for extraction and a *k*_EC_ of 0.45 was used for calculations [31]. Soil respiration was measured with an infrared gas analyzer according to [32] using 30 g (55% WHC) subsamples.

For the measurement of the enzyme activities, two different approaches were used. Firstly, the dehydrogenase activity was measured by the triphenyltetrazolium chloride (TTC) method [29]; secondly, a fluorimetric microplate enzyme assay according to [33], modified by [34], was employed for all other enzymes. Four enzyme substrates based on methylumbelliferone (MUB) and 7-amino-4-methylcoumarin (AMC) were examined, which represent major pathways of C-, N-, and P-cycling in soil: l-leucin-AMC for leucine-aminopeptidase (EC 3.4.11.1), MUB-β-d-cellobioside for ß-cellobiohydrolase (EC 3.2.1.91), MUB-β-d-glucopyranoside for ß-glucosidase (EC 3.2.1.3), and MUB-phosphate for alkaline phosphatase (EC 3.1.3.2) activities. Plates were kept at 30 °C in the dark and then measured in 30-min intervals for 120 min. Fluorescence was measured with a Victor3 MultiLabel Reader (Perkin Elmer, Germany) with an excitation wavelength of 355 nm and an emission wavelength of 460 nm.

### Statistics

The mean values of microbial biomass C, enzyme activity, basal respiration, and metabolic quotient were calculated. The metabolic quotient was calculated as the ratio of soil basal respiration (CO_2_–C) and microbial biomass C (MBC). One-way ANOVA and post hoc Tukey-B test was used to compare between treatments, and *p* < 0.05 was taken as significant cut-off. For statistical analyses, we used SPSS version 22 (SPSS, IBM Corporation, NY).

## Results

### Characterization of the PS-NP suspension prior to application into soils

**Fig. 1:**
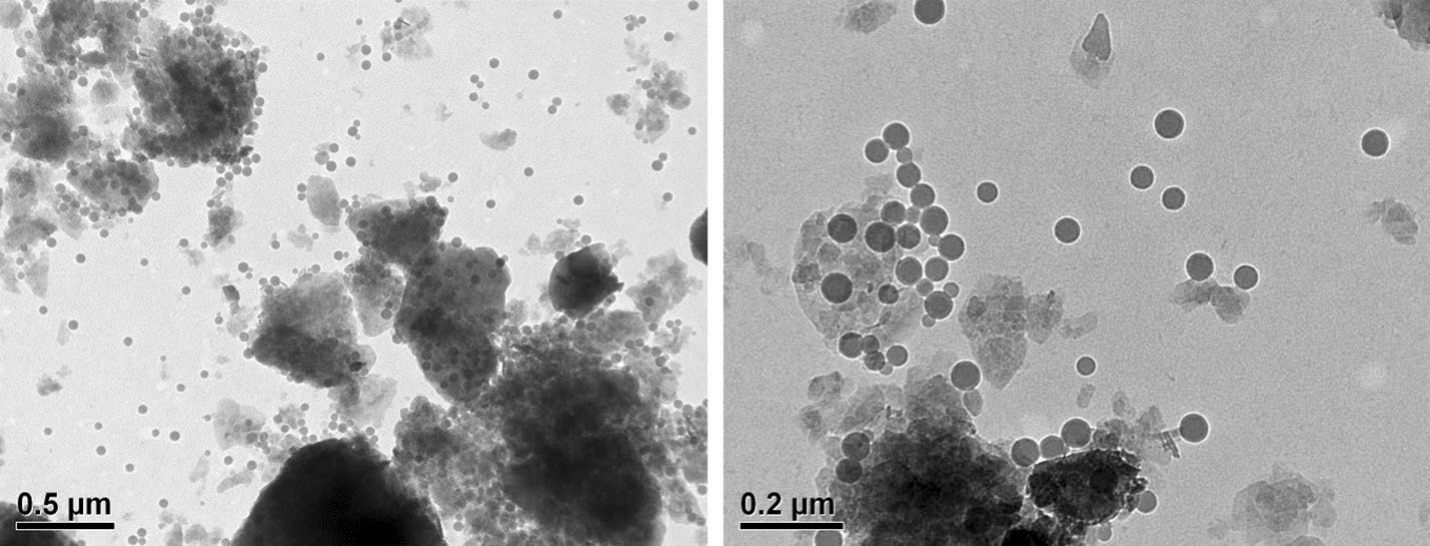
Transmission electron microscope (TEM) image of PS-NP in soil

Polystyrene nanoparticles (PS-NPs) were characterized prior to their use in exposure studies. Characteristics of the PS-NPs were as follows: size (TEM): 32.6 nm ± 11.9 nm; hydrodynamic diameter (DLS, z-average): 69.5 ± 0.5 nm; polydispersity index (PDI) (DLS) = 0.036 ± 0.005; gyration diameter calculated from AF4-MALS (peak maximum) = 46.4 nm ± 0.3 nm; hydrodynamic diameter calculated from AF4-DLS (peak maximum) = 72.3 nm ± 1.2 nm; Zeta-potential: − 43.3 ± 17.5 mV (in ultrapure water). Consistent data from both batch-DLS and AF4-DLS with approx. 4% deviation in hydrodynamic diameter were obtained indicating a narrow particle size distribution in aqueous suspension (see also Fig. 1).

### Occurrence and behavior of PS-NP in soil

The PS-NP could be recognized by its characteristic size and shape as round particles of about 30 nm of intermediate electron density (Fig. 1). The polystyrene was partially attached to the soil particles but between the soil components also single nanoparticles were visible. The PS–NPs did not form aggregates or homo-agglomerates but seemed to agglomerate at the soil particle surface (hetero-agglomeration).

### Microbial biomass and activity

**Fig. 2:**
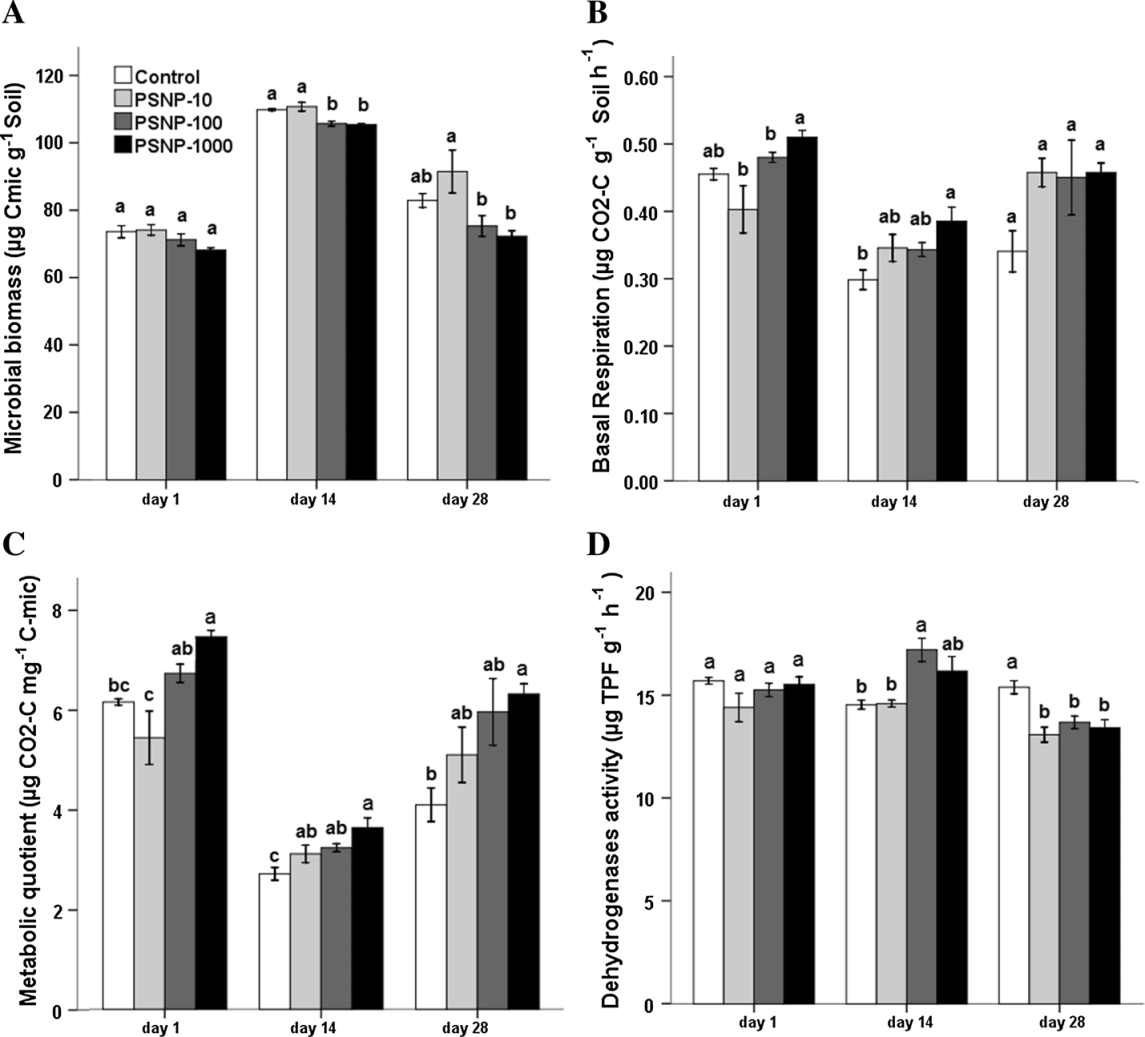
Mean (± SE, *n* = 4) of **A** microbial biomass-C, **B** basal respiration, **C** metabolic quotient, and **D** dehydrogenases activity under different PS-NP concentrations of 10 (PSNP-10), 100 (PSNP-100), and 1000 (PSNP-1000) μg kg^−1^ dry soil at three time points during 28 days of incubation. Significant differences between the treatments are indicated by different letters (ANOVA and post hoc Tukey-B test; *p* < 0.05)

PS**-**NPs showed detrimental effects on the investigated soil microbial biomass and the majority of enzymes. Microbial biomass carbon (MBC) in the treatment of high PS**-**NP concentration application (PS**-**NP-100 and PS**-**NP-1000) was lower than PS-NP-10 and the control throughout the experiment (Fig. 2A). At day 14, the difference was statistically significant; however, at day 28 the decrease for the same treatments was statistically significant for PS-NP-10 but not to the control. When compared to control, low concentration treatment (PS-NP-10) tends to gradually increase MBC with significant increase at day 28.

Basal respiration and metabolic quotient (qCO_2_) showed a trend of increase with increasing PS-NP application compared to control (Fig. 2B). Basal respiration for PS-NP-100 and PS-NP-1000 showed significant increase at day 1 and day 14 as compared to PS-NP-10 and control. All treatments showed an increase in basal respiration compared to control at day 28; however, due to high standard error the increase was not statistically significant. The trend was more prominent for qCO_2_ (Fig. 2C) than for basal respiration, for example, qCO_2_ showed statistically significant difference between the control and the highest concentration (PS-NP-1000) in all treatments.

Microbial activity, measured as dehydrogenase activity, showed no significant difference at day 1 but there was a slight decrease for PS-NP-10 (Fig. 2D). At day 14, despite increased application of PS-NP, dehydrogenase activity increased notably in high concentration treatments, PS-NP-100 and PS-NP-1000, with statistical significance for PS-NP-100 as compared to PS-NP-10 and the control. At day 28 however, PS-NP application showed negative effect on dehydrogenase activity with significant decrease in all treatments as compared to control.

### Functional diversity of exo-enzyme activities

**Fig. 3:**
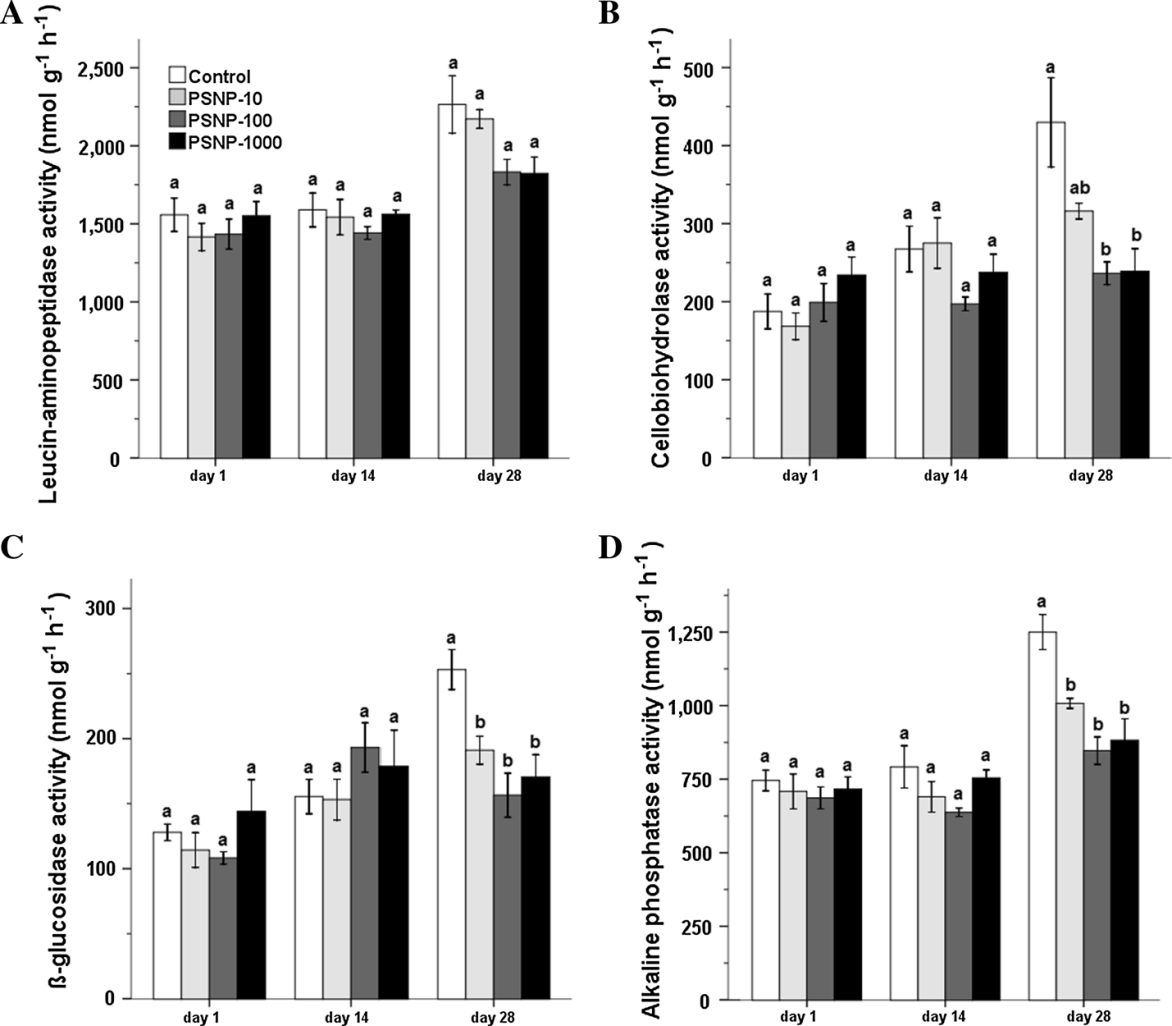
Mean (± SE, *n* = 5) of extracellular enzyme activity **A** leucin-aminopeptidase, **B** cellobiohydrolase, **C** 1,4-β-glucosidase, and **D** alkaline phosphatase under different PS-NP concentrations of 10 (PSNP-10), 100 (PSNP-100), and 1000 (PSNP-1000) μg kg^−1^ dry soil at three time points during 28 days of incubation. Significant differences between the treatments are indicated by different letters (ANOVA and post hoc Tukey-B test; *p* < 0.05)

We found a negative impact for all fluorogenic substrates due to PS-NP application at day 28 with statistical significance for cellobiohydrolase, β-glucosidase, and alkaline phosphatase activities (*p* < 0.05). Leucine-aminopeptidase and alkaline phosphatase activities tended to decrease in all of the treatments throughout the incubation period with pronounced negative effects at the end of the incubation period. When compared to control, at day 1, β-glucosidase and cellobiohydrolase activity decreased at lower concentrations (PS-NP-10 and PS-NP-100) and increased at high concentration (PS-NP-1000). Due to high standard errors, these changes were not statistically significant. However, at day 14, cellobiohydrolase activity decreased at high concentrations (PS-NP-100 and PS-NP-1000) and increased at low concentration (PS-NP-10) while β-glucosidase activity remained high relative to the control (Fig. 3).

## Discussion

We demonstrate for the first time that PS-NP can significantly lower soil microbial biomass and enzyme activities. Soil microbial biomass decreased with increasing application of PS-NP, which was also accompanied by a decrease in most enzyme activities suggesting broad antimicrobial effects of PS-NPs on soil microbiota and enzymes. PS-NPs inherent nano-specific properties, such as small size and high surface area-to-volume ratio, can allow it to closely interact with the microbial cell and the sub-cellular structures that could lead to antimicrobial activities [35]. Moreover, strong sorption affinity of NPs for toxic compounds in soil [36] could have contributed to the toxicity of PS-NPs to soil microbes. Furthermore, although unmodified PS-NPs are generally considered non-toxic, studies have shown that surface charge, size, agglomeration and aggregation of NPs could be altered in environmental media [37, 38]. The surface charge furthermore controls the dispersion and agglomeration of the particles in the soil, which directly determine the bioavailability of the NP and the toxicity to microbiota. A strong aggregation of the NP would lead to bigger particle sizes (micrometers) and a decrease of NP uptake and acute toxicity [39, 40]. However, due to the formation of stable colloids in biological fluids and a low polydispersity index [2], PS-NPs occur as single particles, as also seen in Fig. 1, which can actively affect microbes. Unlike other environments (e.g., marine), soil presents a more complex matrix and nanoparticles are known to interact rapidly with soil particles including minerals and heavy metals, dissolved organic matters and toxic chemicals [3]. Similar interactions could have as well potentially changed PS-NP physiochemical properties to cause an antimicrobial effect.

Interestingly, careful observation at PS-NP-10 reveals a gradual increase in MBC with time that was significant at day 28. At day 1, MBC at PS-NP-10 remained almost the same while enzyme activities, basal respiration rate, and metabolic quotient (qCO_2_) decreased. This suggests a sublethal effect of PS-NP at low concentrations on the first day. Even though the organism-specific effects of NPs are still not clear, selective reduction of certain bacterial populations has been reported. For example, single and multi-walled carbon tubes have been shown to selectively reduce or increase certain phyla and genera of the soil microbial population [41, 42]. Towards the end of the incubation period, the pronounced increase of MBC at PS-NP-10 might be due to an increased PS-NP antimicrobial activity to some microbial genera over time. As a result, dead cells might have provided readily available substrate for the resistant microbes that led to cryptic growth [43].

Our results showed a significant decrease in all enzyme activities at day 28. Since soil dehydrogenases are an integral part of intact cells and are only present in viable cells [44, 45], the decrease in dehydrogenases activity accompanied by the decrease in microbial biomass at day 28 suggests a direct effect of PS-NPs on metabolic activities of soil microorganisms. The exact mechanism of antimicrobial activity can differ and may often have multidimensional effects. Cell membrane disruption by direct contact, physical piercing, and oxidative stresses have been reported as possible mechanisms of antimicrobial activity [46–48]. Using molecular simulations, it has been shown that PS-NPs could easily permeate into lipid membranes, which could severely affect membrane activity and therefore cellular functions [20].

At day 14, however, dehydrogenase activities increased at PS-NP-100 and PS-NP-1000 despite a decrease in MBC. Inconsistent behaviors of dehydrogenases have been reported depending on type [49] and concentration [50] of pollutant, and soil type [51, 52], which has raised a doubt on its reliability [53].

On the other hand, dehydrogenase activity is positively correlated with the number of microorganisms [54] as well as with the organic content [55]. It has been reported that in microbial communities, certain bacteria can excrete beneficial metabolites that can also be used by other local communities [56, 57]. In our result, the activity of β-glucosidase showed similar pattern to dehydrogenase activity throughout the incubation period. The β-glucosidase is an extracellular enzyme that plays an important role in the carbon cycle by producing glucose which is an important energy source for microbes [58]. This may suggest that the change in dehydrogenases activity could be in response to the availability of organic matter, in this case glucose, the byproduct of β-glucosidase rather than microbial biomass.

The activity of β-glucosidase showed similar pattern to dehydrogenase activity throughout the incubation period. The β-glucosidase is an extracellular enzyme that plays an important role in the carbon cycle by producing glucose, which is an important energy source for microbes [58]. Dehydrogenase activity is positively correlated with the number of microorganisms [54] as well as with the organic content [55]. In agreement to the latter, our result suggests that the change in dehydrogenase’s activity could be in response to the availability of organic matter, in this case glucose, the byproduct of β-glucosidase.

Furthermore, the impact of NPs on the microbial community could contribute to microbial physiology and functions, by affecting cell–cell interactions such as genetic exchange and production of secondary metabolites. NPs have been reported to show an inhibitory effect on those metabolites suggesting a potential negative impact of NPs on intercellular interactions in microbial communities [59, 60]. An increased disruption of intercellular interactions may have contributed to the more pronounced changes in MBC and enzyme activities towards the end of the incubation period.

The decrease in activities of extracellular enzymes involved in *N* (leucine-aminopeptidase), *P* (alkaline phosphatase), and C (β-glucosidase and cellobiohydrolase) cycles in the soil at day 28 was consistent with the decrease in MBC and dehydrogenase activity, clearly showing detrimental effects of PS-NPs on soil microbial activity. Extracellular enzymes play a key role in the soil ecosystem function including nutrient cycle and microbial metabolism. Change in the activity of extracellular enzymes has been used to demonstrate the effects of soil contaminants such as heavy metals and antimicrobial agents on soil microorganisms [61, 62]. In our result, the persistent decrease in Leucine-aminopeptidase and alkaline phosphatase throughout the incubation period and the significant decrease in activity of all enzymes at day 28, which was also accompanied by decrease in microbial biomass, suggest that PS-NPs act as stressors to soil microorganisms with a broad impact on nutrient cycling mediated by soil microorganisms. Unlike the activity of other enzymes, β-glucosidase and cellobiohydrolase showed an increased activity at high PS-NP concentrations (PS-NP-1000) at day 1, which persisted for β-glucosidase at day 14. PS-NP might have induced cytotoxicity to some microorganisms, e.g., fungi, which have a high carbon storage mainly due to the chemical composition of their cell wall and are also the predominant source of cellulase enzyme in the soil [27]. The dead microorganisms might have increased the availability of carbon in the soil that caused an increased activity of both enzymes related to the C-cycle. However, the increased activity of cellobiohydrolase at day 1 could be due to its prior presence in soil, while its subsequent significant decline could be due to the negative effect of PS-NP to fungi or related sources. The persistence of β-glucosidase activity at day 14 might be due to the availability of substrates, possibly the byproducts of cellulase activities.

Basal respiration rate showed an increasing trend with increasing PS-NPs application. As dose increased, possible antimicrobial effects of PS-NPs might have selectively decreased the abundance of some microbial genera. As cells die, the surviving bacterial genera may have grown using the readily available remains of the lysed cells that resulted in substrate-induced respiration. In agreement to this, qCO_2_ showed a clear increase with increased application of PS-NPs throughout the incubation period. High values of qCO_2_ indicate a stress-induced respiration where energy is diverted from growth and production to repairing damage due to disturbances [63]. This is a clear indication of the detrimental effects of PS-NPs on soil microbes that could lower substrate use efficiency [64].

## Conclusions

We have demonstrated for the first time that PS-NPs can negatively affect microbial biomass and enzyme activities in soils while basal respiration rate and metabolic quotient increased. We thus suggest that PS-NPs exhibit antimicrobial activity in the soil environment. Furthermore, interaction of PS-NPs with the complex soil environment could contribute to the toxicity of presumably non-toxic PS-NPs. Lack of conclusive evidence on toxicity of nanoparticles in soil is one of the main challenges in environmental risk assessment. These findings provide evidence that PS-NPs pose a potential hazard to soil microorganisms. Future work will investigate a possible change in soil microbial-community composition due to PS-NPs application. Moreover, focus will be laid on the development of analytical strategies, which are able to determine critical physico-chemical properties of the PS-NP such as Zeta potential and agglomeration state directly in or after extraction from soil matrices.

## Additional file

**Additional file 1.** Detailed fractionation conditions for PS-NP.
