# Supplementary material for: Effects of polystyrene nanoparticles on the microbiota and functional diversity of enzymes in soil
Source: Environ Sci Eur. 2018 May 4;30(1):11. doi: 10.1186/s12302-018-0140-6 (PMC5937892; doi:10.1186/s12302-018-0140-6)
Supplement: Supplementary file 1 — Additional file 1. Detailed fractionation conditions for PS-NP. [file 12302_2018_140_MOESM1_ESM.docx]

Additional file

1) DLS measurements

The system used a backscattering angle of 173° at a wavelength of 632.8 nm.

For batch-DLS measurements, the temperature of the measurement cell was set to 25 °C. All measurements were performed in automatic mode, which optimizes attenuation and cell position automatically. The directly obtained intensity-weighted size distribution is converted to a number-weighted distribution by the MALVERN Zetasizer software (version 7.11) according to a refractive index of 1.59.

For the flow-DLS measurements (AF4-UV-MALS-DLS) the cell position was set to 4.2 and the attenuation to 11, which means no attenuation. The measurement time was 3 seconds.

2) Sample preparation

Dilution with ultrapure water, no sonication, thorough mixing for 30 seconds.

3) AF4-UV-MALS-DLS Fractionation System (Postnova Analytics GmbH, Landsberg, Germany) consisting of:

AF2000 MT

PN7520 Solvent Degasser

PN1130 isocratic pump

PN 5300 Autosampler

PN4020 Channel oven

PN3211 UV Detector at a wavelength of 254 nm, R=0.0005

PN3621 MALS

DLS Zetasizer Nano S MALVERN

Injection volume 125 µL at a concentration of 20 mg/L.

4) AF4 separation method and fractogram

The measurements were carried out in triplicate. Further fractionation conditions were as follows: injection volume was adjusted to 125 µl, detector flow with 0.5 mL min^-1^, cross flow 1 mL min^-1^ with a power gradient of 0.2, injection flow 0.2 mL min^-1^ with an injection time of 7 min and a fractionation time of 50 min (see figure 1). A rinse step of 10 min at the end of the fractionation process was used between injections.


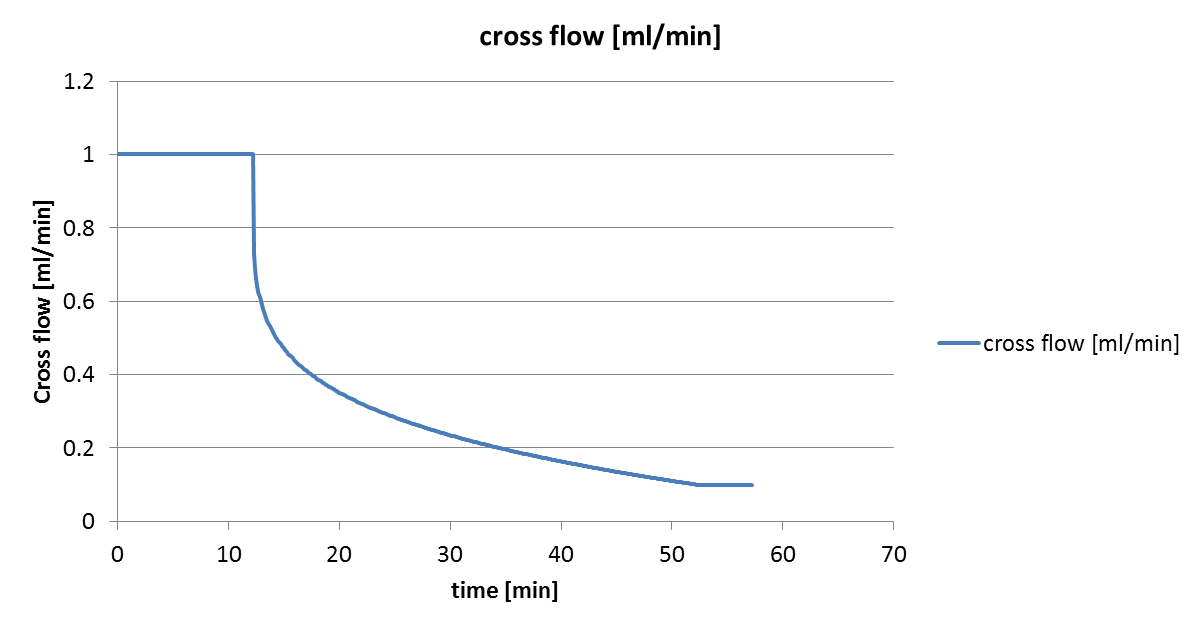


Figure S1: Separation program for AF4-measurements.

5) Data evaluation by AF4-MALS

Gyration diameters were calculated with the sphere model based on the Rayleigh-Debye-Gans Theory within the Postnova AF2000 control software. Detection angles from 12° till 156° (19 of 21 angles applied).

$$\frac{R(\vartheta)}{c K}=MP(\vartheta)$$

with K: contrast factor; ϑ: Scattering angle; R_ϑ_: Rayleigh ratio; c: Concentration of the analyte; λ_0_ the wavelength; M: Molar mass.

$$K=\frac{4\pi^{2}}{\lambda_{0}^{4}N_{A}}{(n_{0}\frac{\mathrm{dn}}{\mathrm{dc}})}^{2}$$

with: n_0_: refractive index of the solvent; dn/dc: refractive index increment of the analyte in solvent: λ_0_: wavelength of the light; N_A_: Avogadro’s number; r: radius of gyration.

$$q=\sin\frac{\vartheta}{2}$$

$$h=rq=r\frac{4\pi n_{0}}{\lambda_{0}}\sin\frac{\vartheta}{2}$$

Sphere model: $P\left( \vartheta\right)=\left[ \frac{3}{h^{3}}\left( sinh-hcosh \right) \right]^{2}$

$$\frac{1}{P(\vartheta)}=1+\frac{16\pi^{2}n_{0}^{2}r^{2}}{3\lambda_{0}^{2}}\sin^{2}(\frac{\vartheta}{2})$$

5) Obtained results

- Batch-DLS


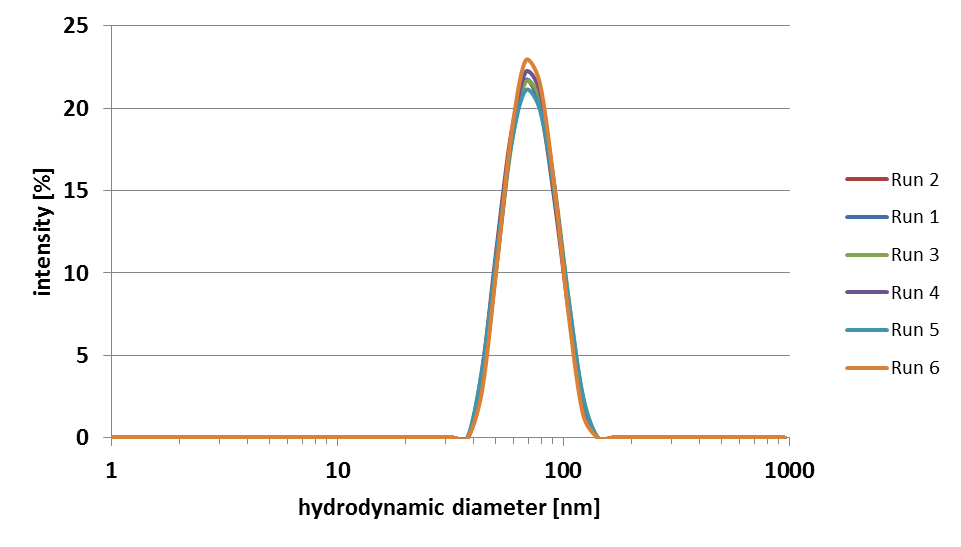


Figure S2: Intensity-weighted size distribution by batch-DLS.


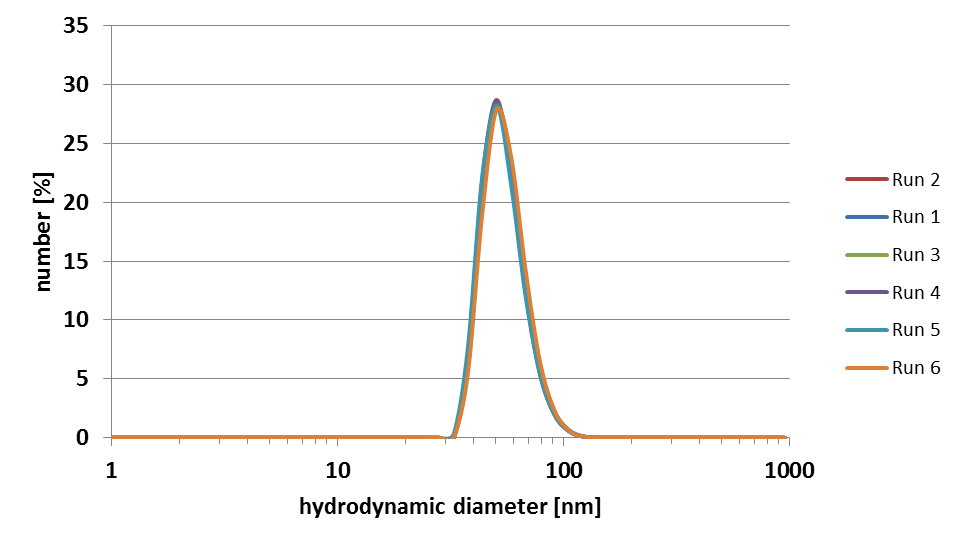


Figure S3: Number-weighted size distribution by batch-DLS.

- AF4-UV-MALS-DLS


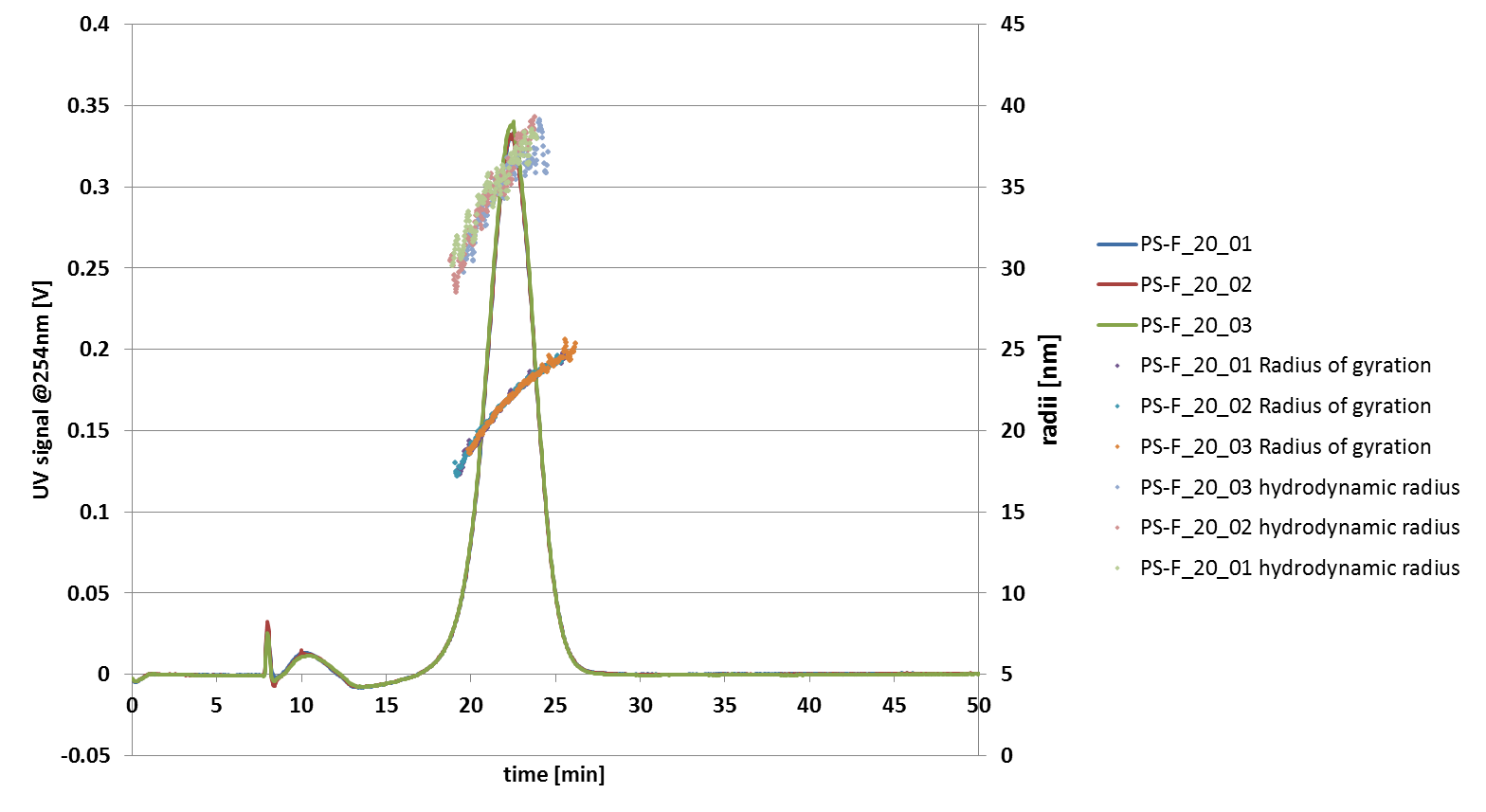


Figure S4: AF4-UV-MALS-DLS measurements corrected by a blank run with radius of gyration and hydrodynamic radius.

Table 1: Results of AF4-UV-MALS-DLS measurements of PS-F Nanoparticles.

|  | **Peak maximum (22.8 min)** | |  |  |  |  |  |
| --- | --- | --- | --- | --- | --- | --- | --- |
|  | **Gyration diameter [nm]** | **Hydrodynamic diameter [nm]** |  |  |  |  |  |
| run 1 | 46.2 | 73.4 |  |  |  |  |  |
| run 2 | 46.5 | 71.2 |  |  | |  |  |
| run 3 | 46.7 | 72.4 |  |  | |  |  |
| **average** | **46.4** | **72.3** |  |  | |  |  |
|  | **0.3** | **1.2** |  |  | |  |  |
|  |  |  |  |  | |  |  |
| **Gyration diameter [nm]** | **46.4 ± 0.3** |  |  | |  |  |  |
| **Hydrodynamic diameter [nm]** | **72.3 ± 1.2** |  |  | |  |  |  |
|  |  |  |  | |  |  |  |


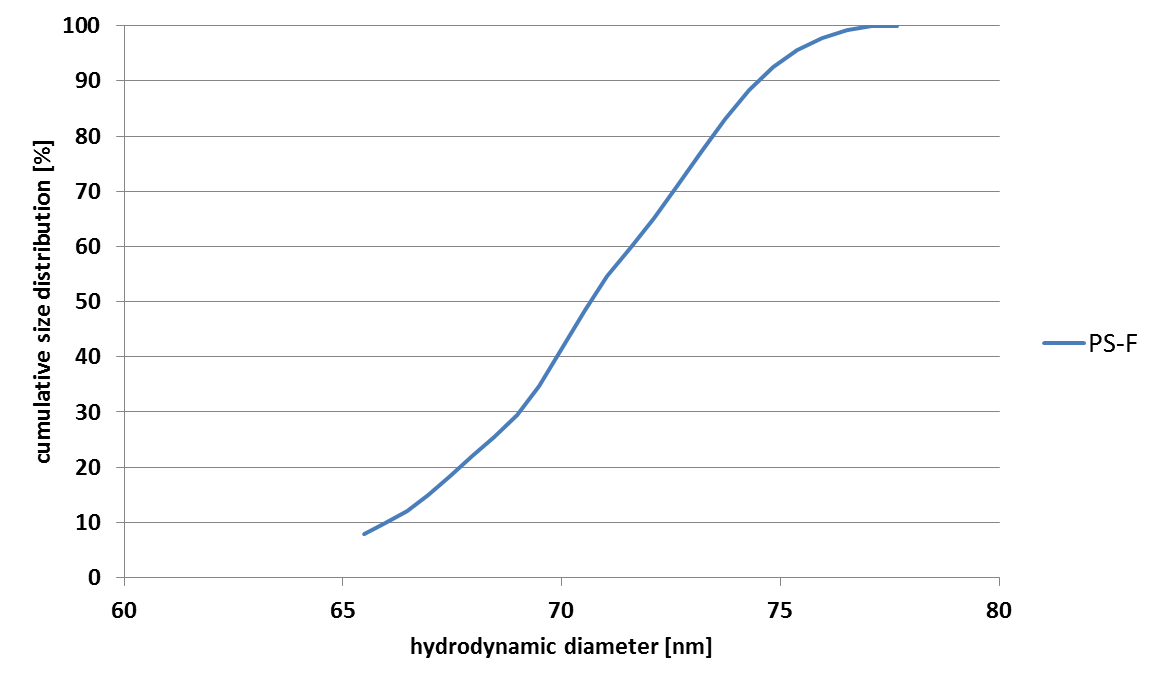


Figure S5: Number-weighted size distribution obtained from AF4-DLS (n=3).


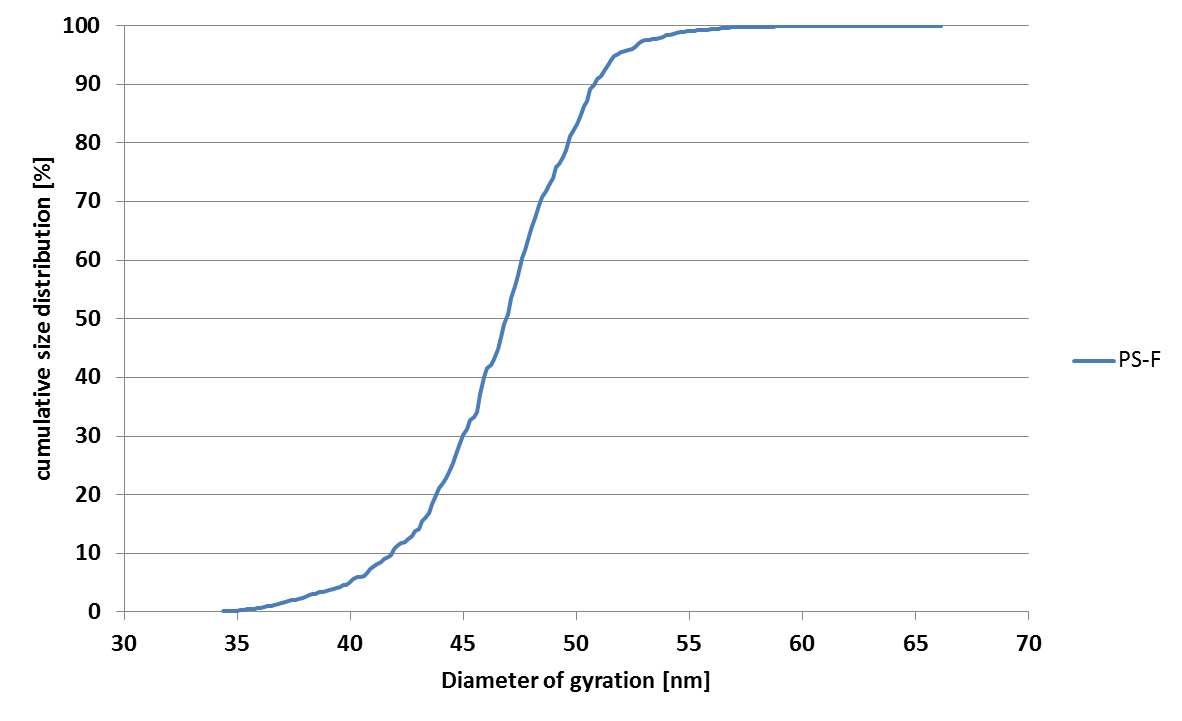


Figure S6: Mass-weighted size distribution obtained from AF4-UV-MALS (n=3).
